# Dystrophic Nails: An Unusual Clue to Recurrent Lymphoma

**DOI:** 10.7759/cureus.28098

**Published:** 2022-08-17

**Authors:** Medha R Cherabuddi, Vijayalakshmi Donthireddy

**Affiliations:** 1 Internal Medicine, Henry Ford Health System, Detroit, USA; 2 Hematology/Oncology, Henry Ford Health System, Detroit, USA

**Keywords:** lymphoma, hodgkin lymphona, diffuse large b cell lymphoma (dlbcl), raynaud phenomenon, nail dystrophy

## Abstract

Nail changes are a well-known phenomenon in T-cell lymphoma but have not been reported as widely in B-cell lymphomas and Hodgkin lymphomas. We describe a patient with a history of diffuse large B-cell lymphoma in a background of nodular lymphocyte predominant Hodgkin lymphoma treated eight years prior who developed new nail changes that were noted on a routine surveillance visit. He had developed symptoms of painful fingertips that became white and required him to wear gloves even in warm weather, suggestive of Raynaud phenomenon. Due to a suspicion of a paraneoplastic phenomenon, a positron emission tomography-computed tomography was obtained, which showed fluorodeoxyglucose avid uptake involving the spleen and retroperitoneal, para-aortic, and right inguinal lymph nodes. Right inguinal lymph node biopsy was non-diagnostic and a splenectomy was performed. Pathology evaluation of the spleen revealed recurrent nodular lymphocyte-predominant Hodgkin lymphoma. Treatment was initiated with rituximab-based systemic therapy. The Beau lines grew out eventually with normal new nail growth and there was an improvement in Raynaud phenomenon after systemic treatment.

## Introduction

Dystrophic nail changes are a well-known phenomenon described in T-cell lymphomas. Nail changes including discoloration, crumbling, onycholysis, Beau lines, ridging, pitting, thinning, etc., have been described in T-cell lymphomas [1, 2]. Reports of nail changes in B-cell lymphomas and Hodgkin lymphomas are limited.

Beau lines are horizontal ridges and indentations that develop on nails. They are indicative of a systemic insult that disrupts nail growth when they appear on multiple nails, such as sickness or chemotherapy. Beau lines are known to appear secondary to Raynaud phenomenon [3, 4]. Raynaud phenomenon has been reported as a paraneoplastic phenomenon in malignancies [5].

We report a case of Raynaud phenomenon and appearance of Beau lines as an unusual clue to systemic lymphoma recurrence.

## Case presentation

A 68-year-old man had initially presented eight years prior to the current evaluation with worsening reflux symptoms and weight loss. A computed tomography scan of the abdomen and pelvis showed adenopathy in the mesentery, upper abdomen, and retroperitoneum including moderate splenomegaly around 14 cm with numerous ill-defined splenic lesions suspicious for lymphoma. A peri-gastric lymph node excisional biopsy revealed areas of typical nodular lymphocyte predominant Hodgkin lymphoma admixed with areas of diffuse large B-cell lymphoma in a T-cell/histiocyte-rich background. He was treated with six cycles of rituximab, cyclophosphamide, adriamycin, vincristine, and prednisone and achieved complete remission.

**Figure 1:**
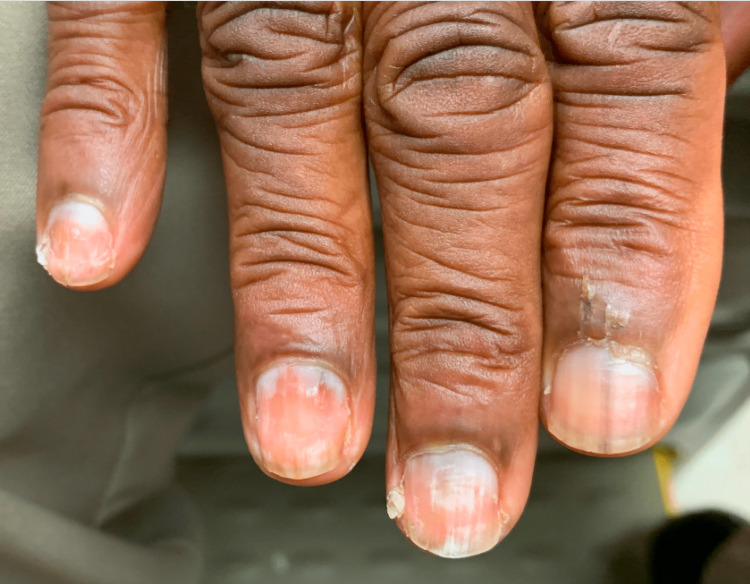
Beau lines with horizontal indentations on nails.

His current presentation stemmed from his report at a routine surveillance visit of having developed new symptoms of painful fingertips that became white along with nail changes that appeared gradually over the preceding year. He reported needing to wear gloves even at room temperature to keep them warm. His nails revealed evidence of Beau lines with horizontal indentations (Figure 1). Autoimmune workup including Scl-70, RNA polymerase III, ENA Ro/La antibodies, creatine phosphokinase (CPK), aldolase and protein electrophoresis was unremarkable, therefore the development of new Raynaud phenomenon raised suspicion for a paraneoplastic phenomenon due to lymphoma recurrence. Fluorodeoxyglucose-positron emission tomography scan was obtained, which showed multiple enlarged para-aortic, retroperitoneal, and right inguinal nodes demonstrating intense hypermetabolism. Scattered areas of focal hypermetabolic lesion were seen throughout the axial and appendicular skeleton involving multiple sites including the proximal right femur, right humerus, multiple ribs, left shoulder, and L1 vertebral body, and two hypodense lesions in the spleen. A right inguinal lymph node biopsy was non-diagnostic. A splenectomy was performed after the completion of pre-splenectomy immunizations.

**Figure 2:**
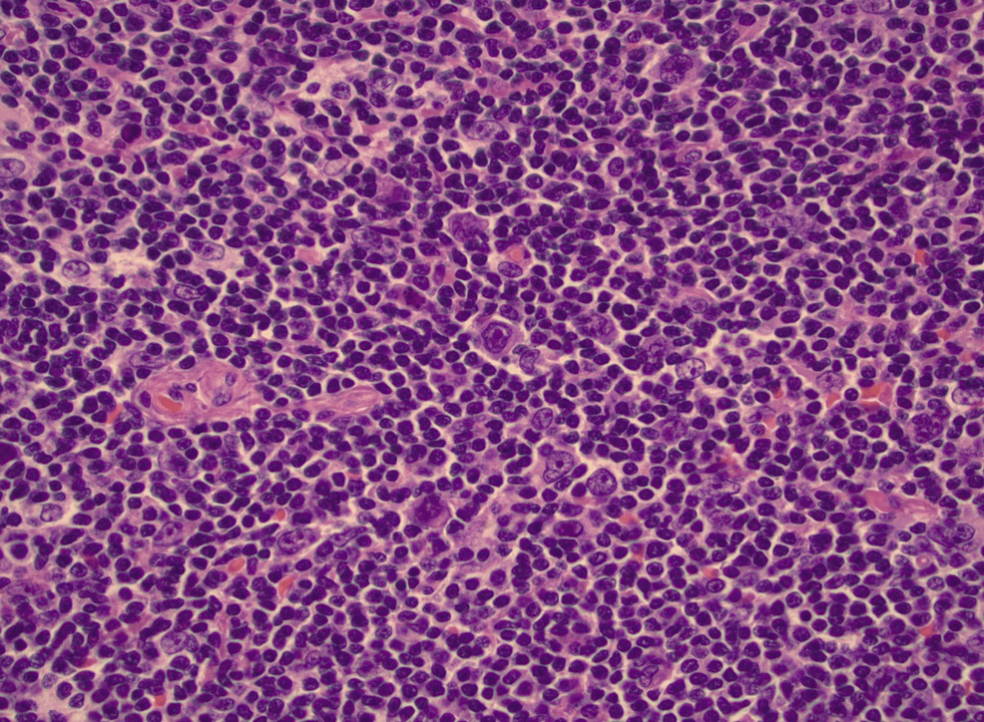
Nodular lymphocyte-predominant Hodgkin lymphoma with hematoxylin and eosin stain in high power field, immunohistochemical staining positive for CD20 and negative for CD30.

**Figure 3:**
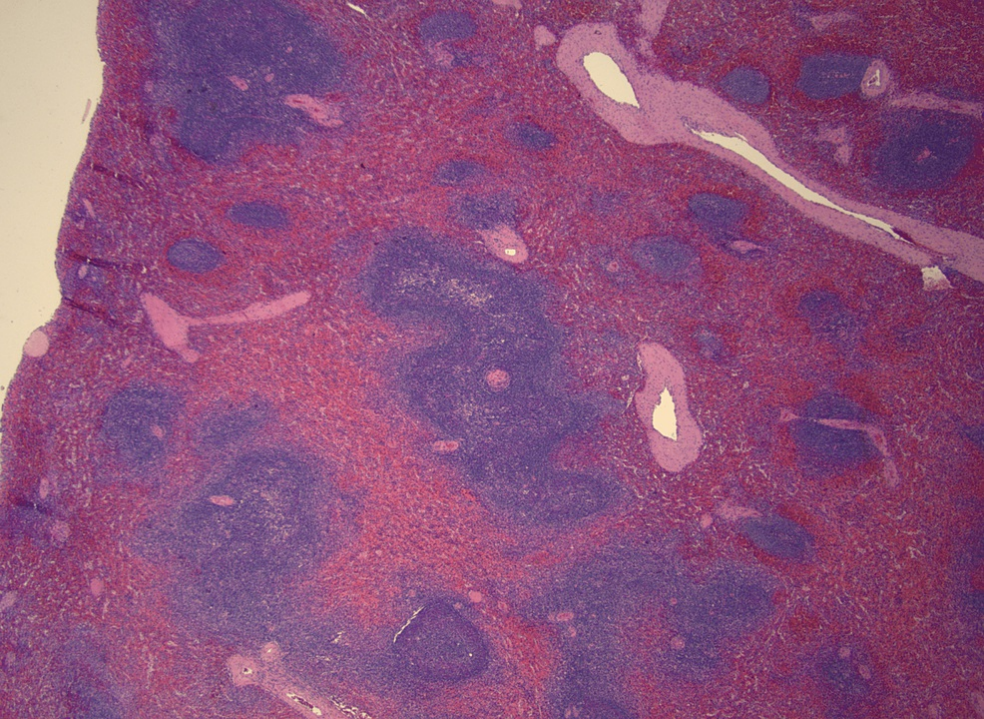
Nodular lymphocyte-predominant Hodgkin lymphoma with hematoxylin and eosin stain in low power field.

Pathologic evaluation of the spleen revealed recurrent nodular lymphocyte-predominant Hodgkin lymphoma (Figures 2, 3).

## Discussion

Nail changes occasionally are a window into the systemic health of the human body. Among the numerous nail changes that are reflective of systemic disease, Beau lines are well described. Beau lines are horizontal indentations and ridges that denote nail plate effects of systemic insults at the time of initial nail formation, such as severe systemic illness, trauma or toxin exposure such as chemotherapy [6]. Beau lines can also be reflective of vascular flow compromise to the tips of digits such as in Raynaud phenomena. Systemic responses to underlying neoplasms manifesting as paraneoplastic phenomena are well-reported, and Raynaud phenomena can result from underlying hematologic or solid organ malignancy [7, 8].

The pathophysiology of Raynaud phenomena is not well understood in the setting of malignancies. Impaired microvasculature, reduced production of vasodilators, and exaggerated response to vasoconstrictors have been postulated [9]. Such vasoconstrictive insult to the actively growing nail plate can cause changes in the deposition of nail matrix leading to Beau nails.

The new occurrence of Raynaud phenomenon or Beau lines should raise suspicion of a systemic process. In our patient, since he had a previous history of lymphoma, there was an immediate evaluation for evidence of disease recurrence. However, in a patient without a prior history of malignancy, a high index of suspicion for an evolving systemic disease is necessary to investigate secondary Raynaud phenomenon before embarking solely on symptomatic therapy.

**Figure 4:**
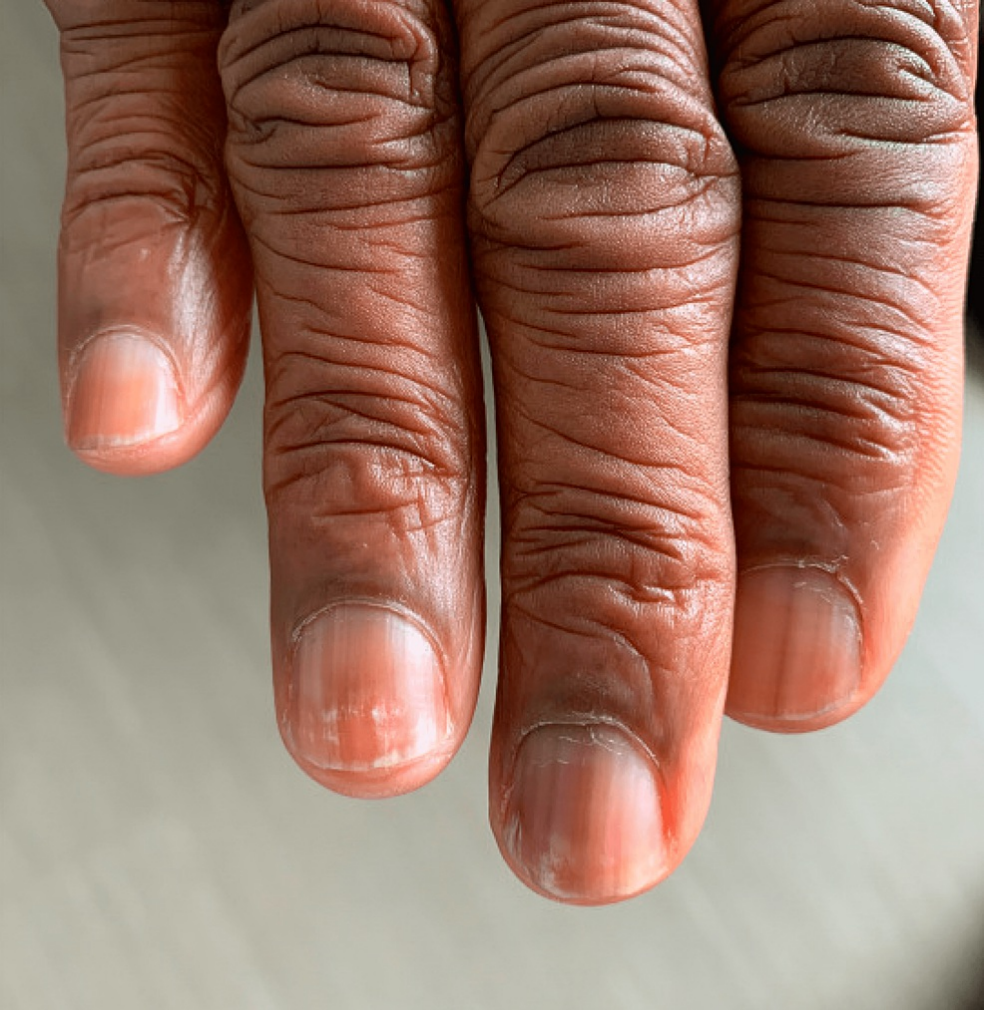
Beau lines grew out eventually with normal new nail growth; picture taken six months after splenectomy.

Our patient was treated for recurrent nodular lymphocyte-predominant Hodgkin lymphoma with rituximab with a good response. The Beau lines grew out eventually with normal new nail growth by six months after treatment (Figure 4). There was an improvement in Raynaud phenomenon around six weeks after treatment.

## Conclusions

In conclusion, the appearance of new dystrophic nail changes should raise suspicion of systemic disease. Beau lines can result from Raynaud phenomenon, which can be a paraneoplastic manifestation of lymphoma and an early sign of recurrent lymphoma.
